# The pro-apoptotic paradox: the BH3-only protein Bcl-2 interacting killer (Bik) is prognostic for unfavorable outcomes in breast cancer

**DOI:** 10.18632/oncotarget.8924

**Published:** 2016-04-22

**Authors:** Vrajesh Pandya, Darryl Glubrecht, Larissa Vos, John Hanson, Sambasivarao Damaraju, John Mackey, Judith Hugh, Ing Swie Goping

**Affiliations:** ^1^ Department of Biochemistry, University of Alberta, Edmonton, Alberta T6G 2H7, Canada; ^2^ Department of Oncology, University of Alberta, Edmonton, Alberta T6G 2H7, Canada; ^3^ Laboratory Medicine and Pathology, University of Alberta, Edmonton, Alberta T6G 2H7, Canada

**Keywords:** breast cancer, BH3-only proteins, Bcl-2 interacting killer, BiK, autophagy

## Abstract

Breast cancer is the leading cause of cancer-associated deaths in women worldwide. Clinical biomarkers give information on disease progression and identify relevant biological pathways. A confounding factor that uncouples markers from disease outcome is the ability of tumor cells to mutate and evade clinical intervention. Therefore, we focussed on apoptotic genes that modulate tumor regression. Using gene and tissue microarray analyses, we identified an association of Bcl-2 interacting killer (Bik) with poor breast cancer prognosis. Bik prognostic ability was independent of Estrogen Receptor/Progesterone Receptor and Her2 status. Additionally, Bik was independent of anti-apoptotic Bcl-2, Bcl-xL, Mcl-1 and Bcl-w suggesting a complex mechanism of tumor promotion identified by Bik high tumors. Bik also stimulates autophagy, which can contribute to enhanced tumor fitness. We found a significant association between the autophagy marker ATG5 and Bik. Combined high expression level of ATG5 and Bik was a stronger predictor of outcome than either alone. Thus, our study identifies Bik as a novel, independent prognostic biomarker for poor outcomes in breast cancer and suggests that Bik-mediated autophagy contributes to disease recurrence.

## INTRODUCTION

Breast cancer is the most prevalent cancer in women worldwide accounting for the highest number of cancer-associated deaths [1–3]. Cancer is a heterogeneous disease with many factors affecting accurate estimation of prognosis, treatment decisions and quality of life. Identification of clinical biomarkers and elucidation of their roles has paved the way for a better understanding of the disease and improved patient survival [4, 5]. Since many unknown factors still determine variable clinical outcomes, we decided to search for additional prognostic markers. We reasoned that due to inherent genomic instability, compensatory mutations would uncouple upstream molecular drivers from clinical outcome. We thus decided to analyze apoptotic markers and in particular focussed on the Bcl-2 family of proteins that are downstream effectors of cell-death or cell-survival decisions [6, 7]. The BH3-only proteins are a subgroup of the Bcl-2 family of proteins [6, 8]. BH3-only proteins have both distinct and overlapping developmental and tissue-specific expression patterns, highlighting both unique and redundant roles in cellular processes [6, 8]. In addition to regulating apoptosis, members of this family also interact with such diverse cellular pathways as autophagy, checkpoint regulation and metabolism [8]. Therefore we examined whether specific BH3-only proteins were prognostic for breast cancer patient outcome and correlated expression with specific biological pathways.

Analysis of gene expression datasets linked to clinical outcomes can identify biomarkers that are significantly regulated at the transcriptional level. Indeed many of the BH3-only proteins are transcriptionally regulated with subsequent effects on apoptotic and autophagic pathways [8, 9]. Genomic stress mediated upregulation of multiple BH3-only genes is dependent on the p53 tumor suppressor [6]. Depending on the nature of stress-stimuli Bid, Bim and Puma are also regulated by FOXO3a while Bim and Puma are subject to E2F-1 dependent regulation [6, 10–12]. Additionally, NF-κB modulates transcriptional activity of Bad and Noxa in squamous cell carcinoma and neutrophils respectively [13]. Although Bik transcription is dependent on p53, various other regulators including TGF-β, E2F, TEF and PARbZIP transcription factors regulate gene expression [14, 15]. Thus multiple pathways that determine cancer cell fate, regulate the gene expression of multiple BH3-only genes.

Importantly, BH3- only protein levels are also regulated at the post-transcriptional level [8, 14]. For instance, Bik is degraded by both proteasomal- and autophagic pathways. [16, 17]. In addition, post-translational modifications of BH3-proteins alter protein function and can act as a signalling switch from apoptosis to cell survival. It is thus important to compare gene expression studies with protein analysis to generate mechanistic models for clinical outcome.

Thus, we investigated the prognostic potential of BH3-only gene expression followed by validation at the protein level. Of five BH3-only genes analyzed, Bik was the only independent prognostic indicator for breast cancer patient recurrence. Bik prognostic value was not dependent on anti-apoptotic gene expression, suggesting that Bik-high tumors were not addicted to anti-apoptotic proteins. However we identified an association between autophagy marker ATG5 and Bik. Our work suggests that Bik may be an indicator of enhanced autophagy and cell survival, contributing to disease recurrence.

## RESULTS

### BH3-only gene expression and breast cancer patient outcome

**Figure 1:**
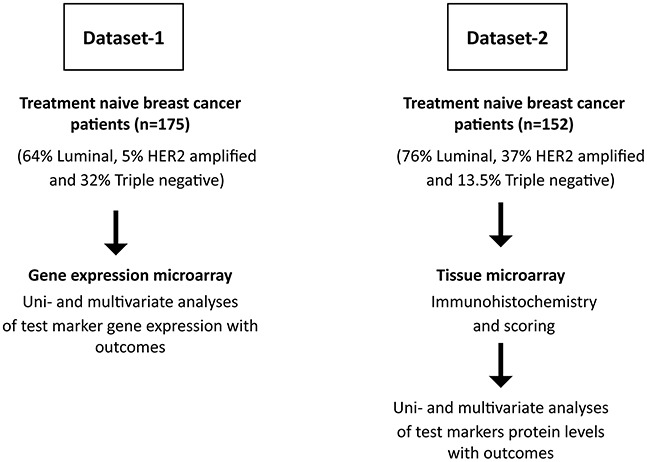
Flow chart depicting selection of patients and experimental scheme Two different patient cohorts were chosen for the study. Treatment-naïve tumor tissue was collected for gene expression studies (Dataset-1) and protein expression studies (Dataset-2). All patients subsequently received standard guideline based therapy.

The BH3-only proteins are comprised of multiple family members with distinct and overlapping roles depending on tissue-type and cellular signals [8]. Whether unique BH3-only proteins regulate breast cancer pathophysiology is not yet clear, and this understanding would aid in efforts to dissect relevant molecular pathways in disease. Therefore, we analyzed a gene expression microarray to identify which, if any, BH3-only genes were associated with clinical outcome. As described in previous studies, this patient cohort contained all pathological subtypes of breast cancer (64% Luminal, 5% HER2 amplified and 32% Triple negative) of variable grade and stage with 49% recurrence and 27% death with complete long-term follow-up after diagnosis (Figure 1) [18, 19]. To identify markers of early recurrence, we assessed five-year disease-free and overall survival. The hazard ratio (HR) indicates the likelihood of relapse or death of the marker-high group versus the marker-low group. The HR values and statistical significance of BH3-only gene expression alongside clinicopathological factors of age, tumor size, mitotic grade, overall grade, vascular invasion, menopausal status, hormone receptor status and Her2 status are shown in Table 1. Of the clinicopathological features, mitotic grade (HR 2.05, p=0.002), overall grade (HR 1.95, p=0.009) and progesterone receptor (PR) status (HR 0.66, p=0.05) were significantly associated with disease-free survival. Gene expression data was retrieved for the BH3-members Bad, Bid, Bik, Noxa and Puma. Univariate Cox regression analysis identified relatively high mRNA levels of only Bid (HR 1.75, p=0.011) and Bik (HR 1.79, p=0.021) to be significantly associated with poor survival outcomes.

**Table 1:** Uni- and multivariate analyses of pathophysiological parameters and BH-3 only gene expression in association with disease-free survival from Dataset-1

Variable	Univariate (Cox)			Multivariate (Cox) - Stepwise		
n=175	HR	95% CI	*p*	HR	95% CI	*P*
**Age>50**	1.10	0.81 to 1.67	0.670		ns	
**Tumor size >2 cm**	1.16	0.76 to 1.78	0.486		ns	
**Mitotic grade**	2.05	1.32 to 3.17	0.002	2.11	1.36 to 3.27	0.001
**Vascular invasion**	1.50	0.97 to 2.33	0.068		ns	
**ER status**	0.72	0.47 to 1.11	0.140		ns	
**PR status**	0.66	0.43 to 1.0	0.050		ns	
**HER2 status**	1.14	0.67 to 1.97	0.628		ns	
**Overall grade**	1.95	1.18 to 3.21	0.009		ns	
**Menopausal status**	0.87	0.57 to 1.33	0.527		ns	
**Bad**	0.73	0.48 to 1.11	0.145		ns	
**Bid**	1.75	1.14 to 2.68	0.011		ns	
**Bik**	1.79	1.09 to 2.92	0.021	1.89	1.16 to 3.09	0.011
**Noxa**	0.73	0.47 to 1.11	0.143		ns	
**Puma**	1.35	0.89 to 2.10	0.166		ns	

ns= non significant

### Bik gene expression is a significant and independent predictor of breast cancer survival

**Figure 2:**
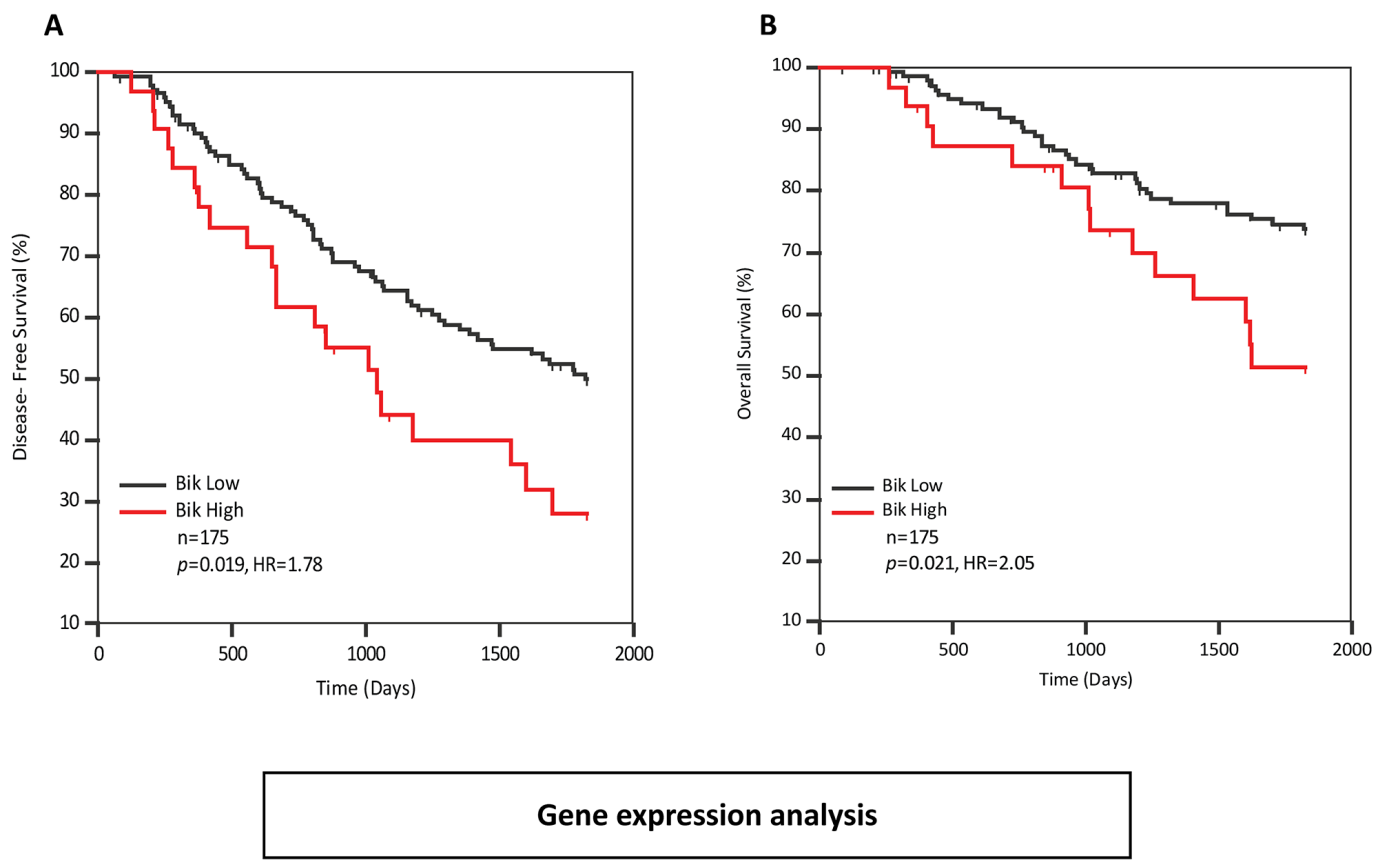
Bik transcript levels are elevated in breast cancer patients with poor survival outcomes Kaplan-Meier survival curves depicting **A.** five year disease-free (HR=1.78, 95% CI: 0.99 to 3.20) and **B.** overall survival (HR= 2.05, 95%CI: 0.96 to 4.37) outcomes of 175 patients based on Bik transcript levels. Patients with low levels of Bik transcript (n=143) were compared to patients with high levels of Bik transcript (n=32). The HR value of greater than 1.0 estimates the predicted risk of poor prognosis.

In order to assess whether Bik or Bid were independently prognostic, we performed multivariate Cox analysis. We carried out stepwise multivariate analysis on all variables associated with recurrence (Bid, Bik, PR, overall grade and mitotic grade). Only Bik and mitotic grade were retained as independent variables. We then performed pairwise multivariate analysis to assess the relationships between variables (Supplementary Table S1). Bid was independent of Bik (HR 1.78, p=0.009) but was dependent on mitotic-grade and overall grade. On the other hand, Bik was independent of mitotic grade, overall grade, PR status and Bid. ROC curve analysis was performed with recurrence as the classification variable to identify an appropriate cut-point (≤1.71) that dichotomized patients into Bik-high and Bik-low groups. Kaplan-Meier analysis of those groups for disease-free survival revealed worse prognosis for Bik high patients with a HR of 1.78 (p=0.019) (Figure 2A). Similar analysis was performed for overall survival. Kaplan-Meier analysis revealed a higher risk of death for Bik high patients with a hazard ratio of 2.05 (p=0.021) (Figure 2B). Thus, elevated Bik mRNA level was an independent prognostic indicator of recurrence and death.

### Elevated Bik protein levels are associated with poor prognosis of breast cancer patients

**Figure 3:**
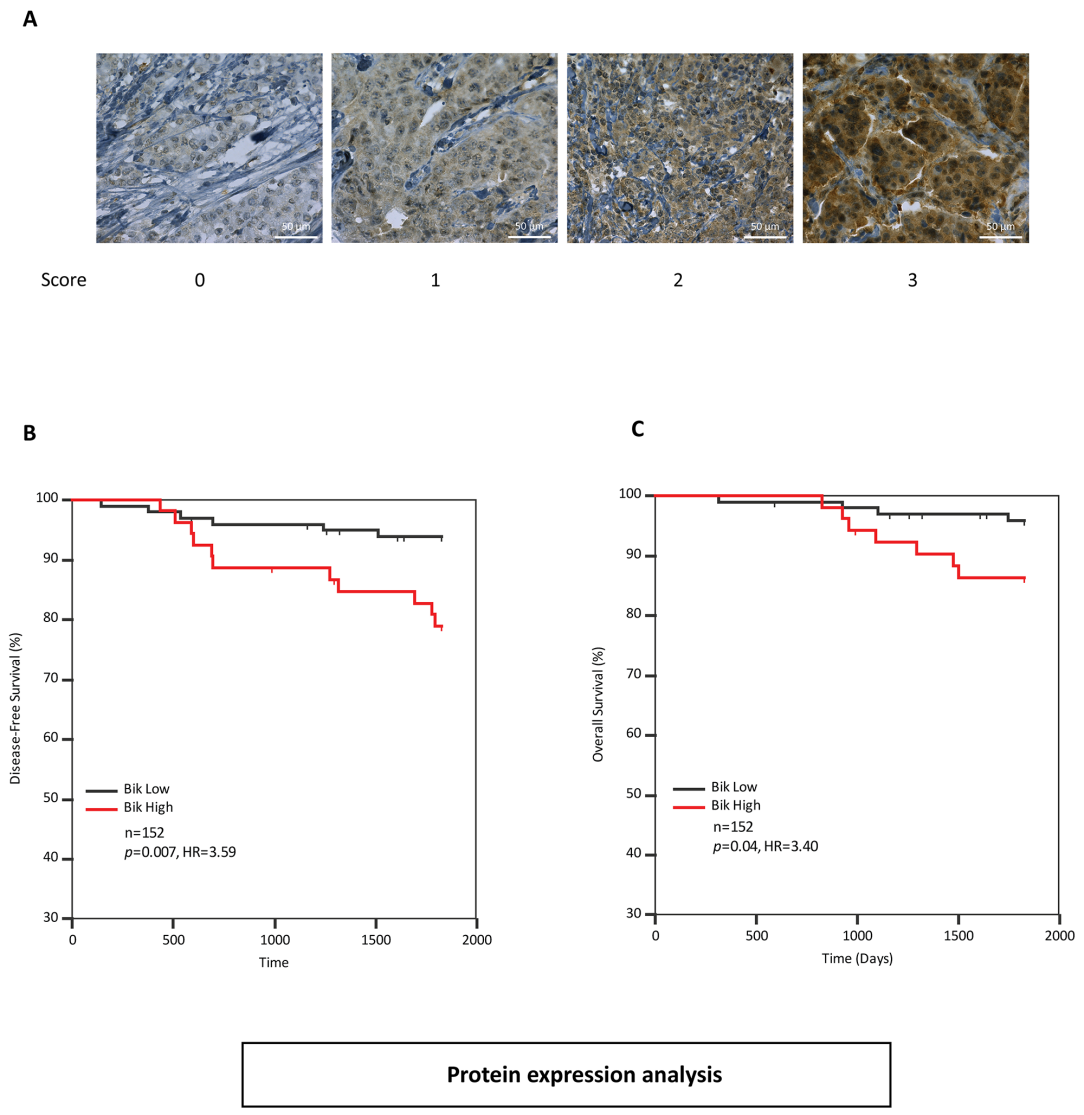
Bik protein levels are elevated in breast cancer patients with poor survival outcomes **A.** Immunohistochemistry analysis of patient tumors stained with anti-Bik antibody. Score values are based on antibody staining intensity (brown) on a scale of 0 to 3. Images are representative of tumor cores with typical score values. Scale bars, 50μm. **B.** and **C.** Kaplan-Meier survival analysis depicting five year disease-free (HR=3.59, 95%CI: 1.32 to 9.82) (B) and overall survival (HR=3.40, 95%CI: 1.10 to 11.69) (C) outcomes of 152 patients based on Bik protein levels in tumor cores. Patients with low levels of Bik protein (n=99) were compared to patients with high levels of Bik protein (n=53). The associated HR value of greater than 1.0 estimates the predicted risk of poor prognosis.

We were curious as to whether Bik protein was also prognostic of outcome. To rigorously test the prognostic value of Bik, we queried a different cohort of patients. We examined a tissue microarray (TMA) from 152 treatment naïve patients (Dataset-2) composed of luminal (76%), HER2 amplified (37%) and triple negative (13.5%) pathological subtypes of variable grade and stage with 11 % recurrence and 16.5 % death (Figure 1). The statistical significance of clinicopathological factors of age, tumor size, mitotic grade, overall grade, vascular invasion, estrogen receptor (ER)/PR and HER2 protein levels are shown in Table 2. Mitotic grade (HR 3.91, p=0.006), overall grade (HR 3.29, p=0.037) and ER status (HR 0.37, p=0.05) were significantly associated with disease-free survival. The TMA was immunostained with an anti-Bik antibody and tumor cells within each tissue core were analyzed for Bik staining. Bik was localized to the cytoplasm and staining intensity for each core was scored on a relative scale between 0-3 by personnel blinded to outcomes (Figure 3A). A Bik score generated from the average value of each associated core was assigned for each patient. ROC analysis identified a cutpoint of ≥1.5. Kaplan-Meier analysis confirmed that high levels of Bik protein were also associated with worse disease-free survival (Figure 3B). Patients with high Bik protein levels had poor disease-free survival with a hazard ratio of 3.59 (p=0.007) compared to Bik low patients. Similarly Bik-high patients had a worse overall survival with a hazard ratio of 3.40 (p=0.04) compared with Bik-low patients (Figure 3C). Multivariate analysis showed that mitotic grade and Bik protein expression were the only two variables that retained significance (Table 2). We then carried out pairwise multivariate analysis to identify dependence of Bik prognostic signature on pathophysiological factors (Supplementary Table S2). Similar to the gene expression analysis, Bik prognostic signature was independent of mitotic grade, overall grade and ER status. Altogether, mRNA and protein analysis identify Bik as a novel independent marker for breast cancer clinical outcomes.

**Table 2:** Uni- and multivariate analyses of pathophysiological parameters and Bik protein level in association with disease-free survival from Dataset-2

Variable	Univariate (Cox)			Multivariate (Cox) - Stepwise		
n=152	HR	95% CI	*p*	HR	95% CI	*p*
**Age>50**	0.36	0.12 to 1.10	0.074		ns	
**Tumor size >2 cm**	1.18	0.44 to 3.09	0.743		ns	
**Mitotic grade**	3.91	1.49 to 10.27	0.006	3.97	1.51 to 10.44	0.005
**Vascular invasion**	0.81	0.31 to 2.14	0.673		ns	
**ER status**	0.37	0.14 to 0.99	0.050		ns	
**PR staus**	0.40	0.16 to 1.05	0.062		ns	
**HER2 status**	0.53	0.14 to 1.95	0.336		ns	
**Overall grade**	3.29	1.07 to 10.09	0.037		ns	
**Menopausal status**	0.58	0.21 to 1.56	0.277		ns	
**Bik**	3.60	1.33 to 9.73	0.012	3.66	1.35 to 9.89	0.011

ns= non significant

### Bik association with poor outcomes is not explained by compensatory increase in anti-apoptotic gene or protein expression

**Figure 4:**
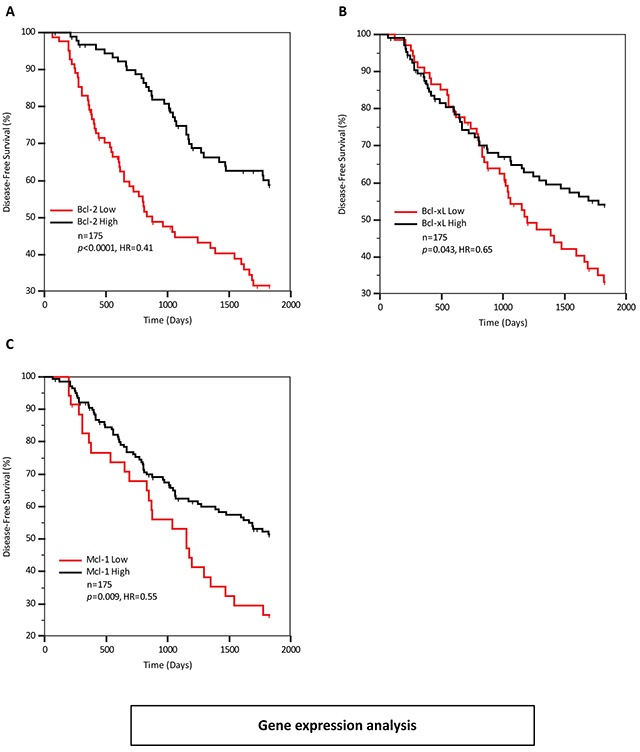
Elevated expression of anti-apoptotic Bcl-2 family members is not associated with poor patient prognosis **A, B** and **C.** Kaplan-Meier survival curves representing disease-free survival outcomes of 175 patients based on Bcl-2, Bcl-xL and Mcl-1gene expression levels respectively.

Bik has a well-documented role as a pro-apoptotic protein, so we were initially surprised that higher levels of Bik mRNA and protein were associated with poor clinical outcomes. A possible explanation was that anti-apoptotic protein levels were upregulated and compensated for Bik activity. For example, Bcl-2 and Bik levels are significantly correlated in multiple myeloma cell lines where Bcl-2 inhibits Bik-induced apoptosis [15]. Therefore, we investigated whether a similar Bik:Bcl-2 association was recapitulated in the clinical samples in our study. We found no correlation with Bik and Bcl-2 gene expression levels (r=−0.02, P=0.79), which did not support a Bcl-2-mediated tumor adaptation model for Bik high tumors. We next examined levels of anti-apoptotic genes in relation to disease-free survival. Data was available for four anti-apoptotic genes (Bcl-2, Bcl-xL, Mcl-1 and Bcl-w). Univariate Cox analysis identified that elevated levels of Bcl-2, Bcl-xL and Mcl-1transcripts were associated with favourable disease-free survival (Table 3). Stepwise multivariate Cox analysis demonstrated that all variables remained significant and were independent of each other. In KM survival analyses (Figure 4 A-C) elevated levels of Bcl-2, Bcl-xL and Mcl-1 were associated with significantly improved disease-free survival with HRs of 0.41, 0.65 and 0.55, respectively. This is opposite to what would be expected if elevated Bcl-2, Bcl-xL and Mcl-1 were indeed inhibiting apoptosis as this would be associated with higher likelihood of recurrence with a HR greater than 1. Finally, using the Bik/Bcl-2 relationship as a model, we stratified patients with respect to their individual Bik and Bcl-2 gene expression levels and looked for correlations between subgroup expression levels and recurrence (Figure-S1). If the tumor adaption model were dominant, we would expect a lower recurrence rate in the Bik-high patient subgroup with low Bcl-2 and presumably unencumbered apoptotic signalling, whereas the Bik-high patients with high Bcl-2 would show significantly higher recurrence rates. However, we see no significant difference between these two subgroups, and in fact the majority of patients show the opposite effect. Thus elevated Bik levels and diminished Bcl-2 levels are both prognostic for poor outcomes, but potentially function in separate pathways.

**Table 3:** Uni- and multivariate analyses of Bik and anti-apoptotic Bcl-2 family members gene expression in association with disease-free survival from Dataset-1

Variable	Univariate (Cox)			Multivariate (Cox) - Stepwise		
n=175	HR	95% CI	*p*	HR	95% CI	*p*
**Mitotic grade**	2.05	1.32 to 3.17	0.002	1.83	1.16 to 2.89	0.010
**Bik**	1.79	1.09 to 2.92	0.021	1.66	1.01 to 2.74	0.047
**Bcl-2**	0.40	0.26 to 0.62	<0.0001	0.47	0.30 to 0.73	0.031
**Bcl-xL**	0.65	0.43 to 0.99	0.045	0.63	0.41 to 0.96	0.031
**Mcl-1**	0.55	0.34 to 0.87	0.010	0.48	0.30 to 0.77	0.003
**Bcl-w**	1.24	0.75 to 2.04	0.399		ns	

ns=non significant

**Figure 5:**
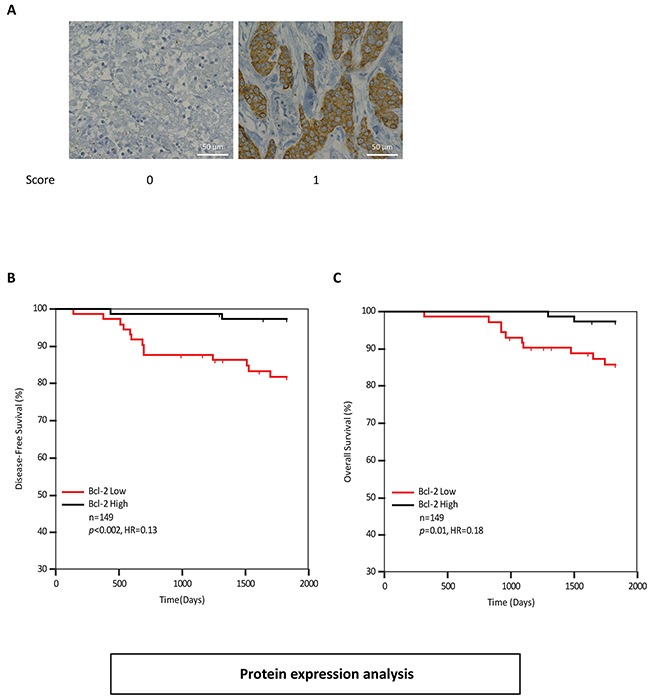
Bcl-2 protein levels are elevated in breast cancer patients with favourable survival outcomes **A.** Immunohistochemistry analysis of patient tumors stained with anti-Bcl-2 antibody. Score values are based on antibody staining intensity (brown) on a scale of 0 and 1. Images are representative of tumor cores with typical score values. Scale bars, 50μm. **B.** and **C.** Kaplan-Meier survival analysis depicting 5 year disease-free (HR=0.13, 95%CI: 0.15 to 0.37) and overall survival outcomes (HR= 0.33, 95%CI: 0.15 to 0.55) of 149 breast cancer patients based on Bcl-2 protein levels in tumor cores. Patients with low levels of Bcl-2 protein (n=73) were compared to patients with high levels of Bcl-2 protein (n=76). The associated HR value of less than 1.0 estimates the predicted risk of poor prognosis.

We next examined Bcl-2 protein levels in the previously described TMA from Dataset-2. Tumor cells within each core were assessed for Bcl-2 protein levels (Figure 5A). ROC analysis identified a cutpoint of >0. Kaplan-Meier analysis confirmed that high levels of Bcl-2 protein were associated with improved disease-free survival (Figure 5B). Patients with high Bcl-2 protein levels had a higher recurrence-free survival with a hazard ratio of 0.13 (p<0.002) compared to Bcl-2 low patients. Similarly Bcl-2-high patients had a higher overall survival with a hazard ratio of 0.18 (p=0.01) compared with Bcl-2-low patients (Figure 5C). Additionally, we performed a multivariate Cox analysis of Bik and Bcl-2 protein levels; Bik and Bcl-2 were found to be independent variables (Table 4). Altogether, our analyses indicate that Bik prognostic value is independent of anti-apoptotic protein levels, suggesting that a non-apoptotic activity of Bik contributes to poor clinical outcomes.

**Table 4:** Uni- and multivariate analyses of Bik and Bcl-2 protein levels in association with disease-free survival from Dataset-2

Variable	Univariate (Cox)			Multivariate (Cox) - Stepwise		
n=139	HR	95% CI	*p*	HR	95% CI	*p*
**Bik**	2.99	1.07 to 8.35	0.038	3.52	1.25 to 9.88	0.017
**Bcl-2**	0.21	0.06 to 0.74	0.016	0.18	0.05 to 0.65	0.009

### High Bik and ATG5 levels are associated with worse patient outcome

**Figure 6:**
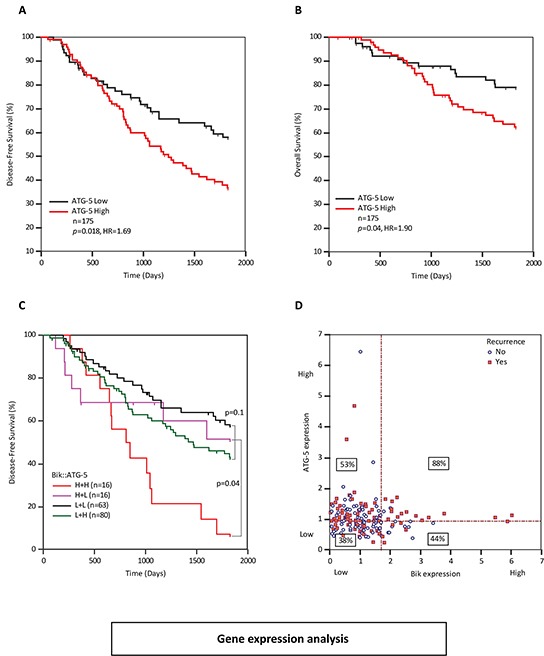
ATG5 transcript levels are elevated in breast cancer patients with poor survival outcomes **A.** and **B.** Kaplan Meier survival curves representing disease-free (HR=1.69, 95%CI: 1.11 to 2.65) survival and overall survival (HR=1.90, 95%CI: 1.11 to 3.41) outcomes of 175 BC patients based on ATG5 mRNA levels. **C.** Paired KM curves demonstrating individual or combined effects of high ATG5 and Bik expression levels on recurrence-free survival of patients. **D.** Scatter-plot comparing gene expression levels of ATG5 and Bik per patient with respect to disease recurrence. Horizontal and vertical dotted lines indicate dichotomizing score cut-points as determined by ROC analysis. Percentage values in rectangles indicate percent of patients that recurred within each quadrant.

We sought to identify a tumor-promoting pathway that accounts for the poor prognosis in Bik high patients. In addition to stimulating apoptotic pathways, Bik facilitates autophagy [20–22] and enhanced autophagy is associated with poor clinical outcomes [23–25]. Thus, using the gene expression microarray as a screening tool, we looked for a correlation between high Bik expression levels and markers of autophagy. Four autophagy gene sets (ATG5, ATG7, Beclin-1 and p62) were captured in the gene expression microarray (Supplementary Table S3). ATG5, ATG7 and p62 showed clear association of high autophagy flux with poor patient outcomes. ATG5 showed the best ROC values so we analyzed ATG5 further. The ATG5 gene product is an E3 ubiquitin ligase involved in elongation of autophagosomal membranes that facilitates tumor survival in nutrient deprivation [26, 27]. Overexpression of ATG5 is associated with aggressive tumors in squamous cell carcinoma, colorectal and prostate cancers and chemoresistant tumors in gastric cancer [26–29]. To examine dependence of Bik and ATG5, we performed ROC curve analysis and identified a cut-point (>0.939) that dichotomized patients into ATG5-high and -low groups. Kaplan-Meier survival analysis of patients with respect to ATG5 gene expression levels demonstrated that patients with high levels of ATG5 mRNA had a disease-free survival HR of 1.69 (p=0.018) compared to ATG5-low patients (Figure 6A). As well, ATG5-high patients had a lower overall survival with a HR of 1.90 (p=0.04) compared to ATG5-low patients (Figure 6B). In order to dissect the relationship between Bik and ATG5, we performed multivariate Cox analysis and found that Bik and ATG5 were independent variables (p=0.02 and p=0.02 respectively). We performed a bivariable KM analysis in order to further examine potential relationships between Bik and ATG5 (Figure 6C). We found that while ATG5-high was a stand-alone significant prognostic indicator (Figure 6A), Bik-high was significant only when ATG5 was also high (Figure 6C), suggesting that the mechanism whereby high Bik contributes to poor outcomes may be through an ATG5-dependent autophagy pathway. To interrogate this further, we generated a scatter plot to correlate patients with respect to their individual Bik and ATG-5 gene levels and examined recurrence rates for each subgroup (Figure 6D). We observed that Bik-high patients with high levels of ATG5 mRNA had 88% recurrence (p=0.009) compared to a 44% recurrence rate in the Bik high/ATG5-low patient subgroup. Of the Bik-low patient subgroups, there was no significant difference in recurrence rates relative to ATG-5 levels. Collectively, this data supports a mutual association of Bik and ATG5 in determining patient outcome.

## DISCUSSION

We identified that the BH3-only protein Bik was a novel prognostic marker for breast cancer. Bik levels were prognostic for disease-free and overall survival in two independent cohorts of primary breast cancer patients. Importantly, elevated Bik levels were associated with poor outcomes (average HR= 2.75). This result suggested that Bik may have tumor-promoter activity. However, there is substantial evidence that Bik is a tumor-suppressor. For example, the Bik gene was mutated in peripheral B-cell lymphomas, deleted in gliomas, head and neck and colorectal cancers or silenced in renal cell carcinomas [14, 30–33]. Additionally, a genetically-engineered mutant of Bik (BikDD) induced apoptosis in pancreatic, breast and colon cancer models resulting in tumor clearance [34–37]. On the other hand, Bik expression was elevated in breast, pancreas, multiple myeloma and colon cancers, suggestive of a tumor-promoting role for Bik [38–41]. These studies, however, did not correlate Bik levels to outcome. As far as we know, our study is the first to report that high Bik transcript and protein levels correlated with poor outcomes.

How Bik association with poor clinical outcome is manifested at the molecular level is unclear. Bik could either be a marker of aggressive tumors that have evolved anti-apoptotic strategies, or Bik could have a direct tumor-promoting role. To address the first point, it is possible that tumors upregulate Bcl-2 expression. This compensatory mechanism to ablate apoptotic pathways is well-documented in multiple myeloma and non-small cell lung cancer (NSCLC) tumor models [15, 42]. Of particular interest, Lu et al reported that poorly surviving non-small cell lung cancer (NSCLC) patients had high Bik expression and compensatory increase in anti-apoptotic protein Bcl-2 led to tumor adaptation [14, 42]. Contrary to their findings, in breast cancer we found that Bik and Bcl-2 were independent variables and the increase in Bik expression was not correlated with expression levels of 4 anti-apoptotic members of the Bcl-2 family. Furthermore, the anti-apoptotic genes Bcl-2, Bcl-xL and Mcl-1 were all independently prognostic of favourable outcome, which argues against a simple anti-apoptotic/tumor-promoting role for these genes. In addition, loss of apoptosis in the face of Bik elevation may involve dysregulation of downstream apoptotic effectors and their regulators. Additionally, recent studies report BH3-only proteins can lead to tumorigenesis by compromising mitochondrial integrity, facilitating low caspase activation and chronic genomic damage [43]. It is possible that Bik-expressing tumors have greater genomic instability driving tumor adaptation and the development of more aggressive subtypes of cancer. Taken together, our data suggests that a complex network may be at play.

Variable clinical outcomes have correlated with anti-apoptotic protein expression dependent on tumor type. Negative outcome associations are seen in Hodgkin's lymphoma, myeloid leukemia and follicular lymphoma, and positive outcome associations are seen in NSCLC, breast and liver cancers [44–47]. In our case, Bcl-2 levels were associated with good outcome, and this is in agreement with other studies [44, 48, 49]. While Bcl-2 can also inhibit cell proliferation and autophagy [21, 50–53], the mechanism behind the positive prognostic signature of the anti-apoptotic genes in our study, is not clear.

**Figure 7:**
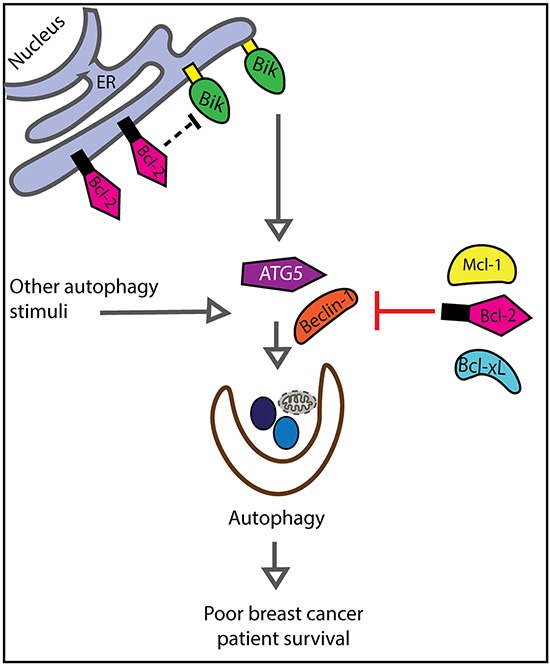
Model describing relationships between Bik, Bcl-2 and ATG5 with respect to patient survival We propose that Bik stimulates autophagy and Bcl-2 inhibits autophagy. Thus, Bik and Bcl-2 function in two separate yet converging pathways associated with poor patient prognosis.

It is possible that Bik stimulates tumor-promoting autophagy. Bik stimulates autophagy through Bcl-2-dependent and -independent mechanisms. In the first case, Bik indirectly stimulates autophagy by ablating Bcl-2:Beclin-1 interactions [21]. Specifically, Chang et al reported that Bcl-2 interaction with the ER-resident protein, Naf-1 was crucial for inhibition of Beclin-1-mediated autophagy and Bik suppressed this Bcl-2/Naf-1 interaction [21]. In the case of the Bcl-2-independent autophagy pathway, ectopic expression of Bik induced autophagy in Bcl2^−/ −^ MEFs [20]. Bik was a target of autophagy and also actively contributed to autophagy induction [16]. Finally, silencing of endogenous Bik expression in the breast cancer cell line MDA-MB-231 downregulated crucial components of autophagic machinery [22]. We found that high Bik expression in breast cancer patients was significantly correlated with increased levels of the autophagy marker, ATG5. ATG5 is a prognostic indicator of squamous cell carcinoma, prostate, and colorectal cancers relapse [26, 29, 54]. This is consistent with a model whereby Bik induced-autophagy promotes breast cancer relapse (Figure 7). Additionally, we speculate that anti-apoptotic Bcl-2-like proteins act downstream of Bik and/or directly antagonize Bik to inhibit autophagic processes. This may explain in part, why elevated Bcl-2 is associated with favourable outcomes. This multiple interaction network of Bik, Bcl-2 and the marker of autophagy ATG5 may then have subset interactions, and thus retain statistical independence. As well, we expect that other signals stimulate ATG5 and autophagy independently of Bik, again resulting in the retention of the independent prognostic value of Bik and ATG5. Although we propose that Bik promotes tumor survival by facilitating autophagy, other possible mechanisms could lead to Bik prognostic association with poor outcomes.

Altogether, multiple models of Bik-induced tumor survival signaling could be at play and lead to the observation of poor clinical outcome. Our study results give rationale to investigate non-canonical roles of Bik related to tumor growth. In addition, exploration of interactions of Bik with autophagy and clinical response in breast cancer patients could set the stage for novel therapeutic regimens in the future.

## MATERIALS AND METHODS

### Reagents

Bik antibody (sc-1710) used for IHC was obtained from Santa Cruz, USA. Bcl-2 antibody (M0887) used for IHC was from Dako, USA.

### Gene expression and tissue microarray analyses

Pre-treatment tumor samples were accessed from the Alberta Cancer Research Biobank/CBCF Tumor Bank. Patient information was collected under research ethics board approval (HREB Biomedical). All patients received guideline based standard therapy. Gene expression data from 175 primary breast cancer patients was determined from analysis of Agilent Whole Human Genome Arrays and has been described previously [18]. The messenger RNA (mRNA) expression data sets were deposited (GEO accession ID GSE22820) in a publicly accessible database. This cohort is referred to as Dataset-1 in this study. For Dataset-2, tissue microarrays (TMA) were generated from formalin fixed tumor samples obtained for 152 primary breast cancer patients as described previously [55]. Briefly, the TMA was prepared by punching at least two representative tumor cores from pre-treatment formalin fixed paraffin embedded (FFPE) blocks and embedding them in recipient paraffin blocks. Glass TMA slides were generated. TMAs were deparaffinized in xylene and serially rehydrated in 95% to 0% ethanol solution. Epitope retrieval was performed by microwaving in citrate buffer (10mM citrate, 0.05% Tween-20; pH 6.0). Immunostaining on these TMAs was performed using anti-Bik antibody (1:300; Santa Cruz Biotechnology, Inc. USA) and anti-Bcl-2 antibody (1:300; Dako, USA). Dako EnVision+System-HRP (Dako, USA) system was used for signal amplification with DAB substrate.

### Scoring and quantification of immunohistochemical staining

The scoring of the immunostained TMA was performed in an outcome blinded fashion according to training and guidelines from the study's breast pathologist. Images were acquired at 40X magnification using a Carl Zeiss Axio Observer microscope (Carl Zeiss, Germany)

### Statistical analysis

Statistical analysis was performed using MedCalc version 15 (Ostend, Belgium). Receiver operator characteristic (ROC) curve analysis was used as a measure of sensitivity versus specificity for determining the optimum cutpoint value for gene expression and TMA scores. Disease-free and overall survival were calculated by Kaplan-Meier analysis. Significant differences between KM curves were measured by log-rank test. Uni- and multivariate analyses for recurrence or death was carried out using Cox regression methods.

## SUPPLEMENTARY FIGURE AND TABLES


