# Supplementary material for: The pro-apoptotic paradox: the BH3-only protein Bcl-2 interacting killer (Bik) is prognostic for unfavorable outcomes in breast cancer
Source: Oncotarget. 2016 Apr 22;7(22):33272–85. doi: 10.18632/oncotarget.8924 (PMC5078093; doi:10.18632/oncotarget.8924)
Supplement: Supplementary file 1 [file oncotarget-07-33272-s001.pdf]

SUPPLEMENTARY FIGURE AND TABLES

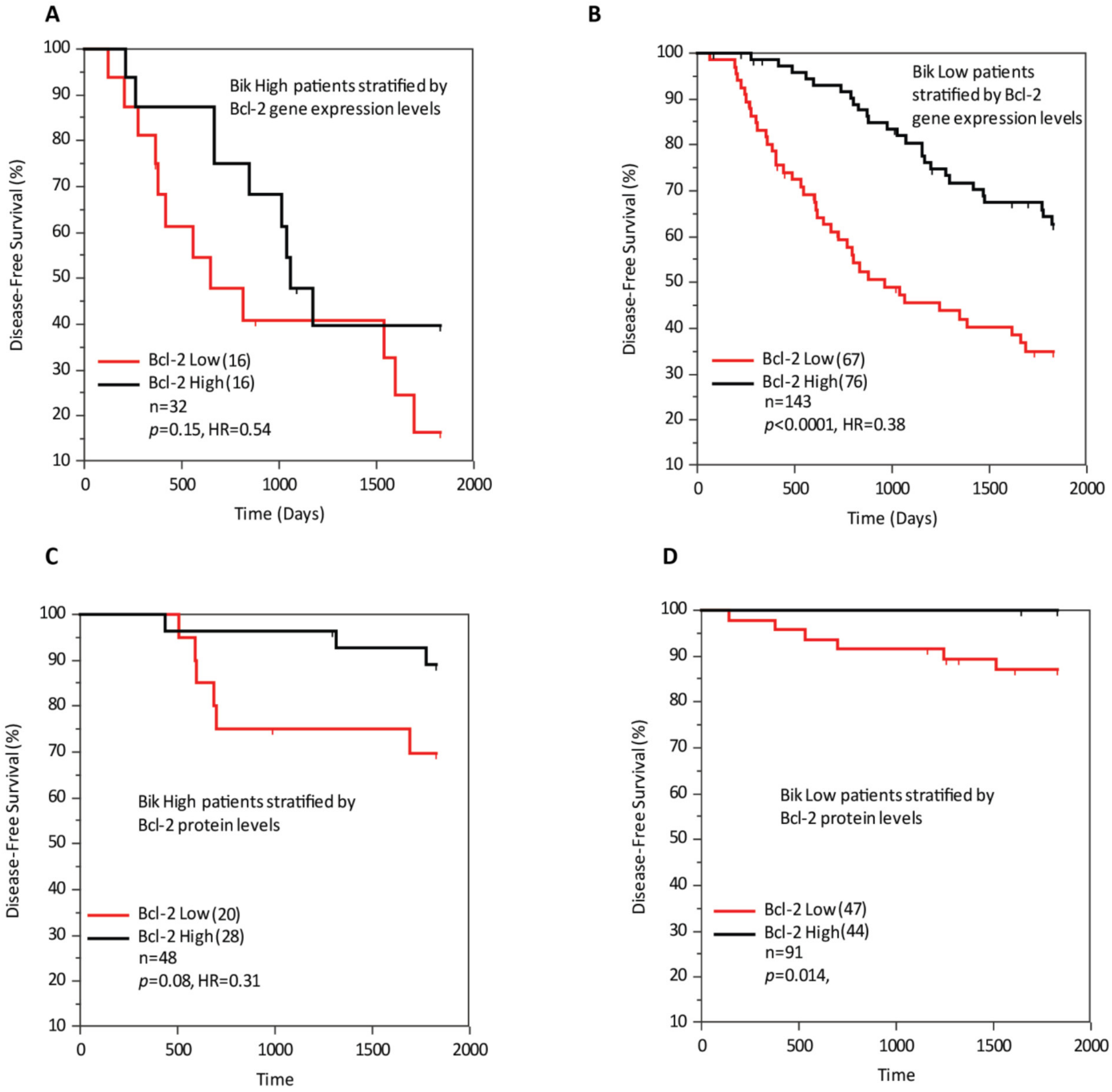

Supplementary Figure-S1 Kaplan-Meier survival curves depicting five year disease-free survival outcomes of A. Bik high (n=32) patients stratified into Bcl-2 high and low groups **B.** Bik Low (n=143) patients stratified into Bcl-2 high or low groups. Outcomes of patients are based on Bik and Bcl-2 transcript levels. Kaplan-Meier survival curves depicting five year disease-free survival outcomes **C.** of Bik high (n=48) patients stratified into Bcl-2 high and low groups and **D.** Bik Low (n=91) patients stratified into Bcl-2 high or low groups. Outcomes of patients are based on Bik and Bcl-2 protein levels.

Supplementary Table S1: Pairwise multivariate analysis of Bik and Bid gene expression against significant pathophysiological variables in association with disease-free survival from Dataset-1

Variable	Multivariate (Cox) - Stepwise		
n=175	HR	95% CI	*p*
Mitotic grade	2.11	1.36 to 3.27	0.001
Bik	1.89	1.16 to 3.09	0.011
Bid	1.78	1.65 to 2.74	0.009
Bik	1.84	1.12 to 3.00	0.016
Bik	1.93	1.20 to 3.20	0.009
PR status	0.61	0.40 to 0.94	0.024
Bik	1.86	1.14 to 3.05	0.001
Overall grade	2.00	1.21 to 3.30	0.007
Mitotic grade	2.04	1.32 to 3.17	0.002
Bid			ns
Overall grade	1.95	1.18 to 3.21	0.009
Bid			ns
PR status			ns
Bid	1.74	1.14 to 2.70	0.01

ns= non significant

Supplementary Table S2: Pairwise mutivariate analysis of Bik against significant pathophysiological variables from Dataset-2

Variable	Multivariate (Cox) - Stepwise		
n=152	HR	95% CI	*p*
Bik	3.66	1.35 to 9.89	0.011
Mitotic grade	3.97	1.51 to 10.4	0.005
Bik	3.54	1.31 to 9.57	0.013
Overall grade	3.22	1.05 to 9.90	0.040
Bik	3.6	1.33 to 9.73	0.010
ER-status			ns

ns= non significant

Supplementary Table S3: Univariate analysis of autophagy marker gene expression in association with disease-free survival from Dataset-1

Variable	Multivariate (Cox) - Stepwise		
**n=175**	**HR**	**95% CI**	***p***
**ATG5**	1.69	1.1114 to 2.5795	0.018
**ATG7**	2.38	0.8223 to 6.8692	0.016
**Beclin-1**	0.56	0.3488 to 0.9042	0.008
**p62**	0.17	0.038 to 0.7882	<0.001
